# Increased insulin-like growth factor 1 production by polyploid adipose stem cells promotes growth of breast cancer cells

**DOI:** 10.1186/s12885-018-4781-z

**Published:** 2018-09-05

**Authors:** Roberta Fajka-Boja, Annamária Marton, Anna Tóth, Péter Blazsó, Vilmos Tubak, Balázs Bálint, István Nagy, Zoltán Hegedűs, Csaba Vizler, Robert L. Katona

**Affiliations:** 1 Biological Research Centre of the Hungarian Academy of Sciences, Institute of Genetics, H-6726 Temesvári krt. 62, Szeged, Hungary; 2 Biological Research Centre of the Hungarian Academy of Sciences, Institute of Biochemistry, H-6726 Temesvári krt. 62, Szeged, Hungary; 3 Creative Laboratory Ltd, H-6726 Temesvári krt. 62, Szeged, Hungary; 4 Biological Research Centre of the Hungarian Academy of Sciences, Institute of Biophysics, H-6726 Temesvári krt. 62, Szeged, Hungary

**Keywords:** Adipose stem cells, Breast cancer, Insulin-like growth factor 1, Polyploidy, Transcriptome

## Abstract

**Background:**

Adipose-tissue stem cells (ASCs) are subject of intensive research since their successful use in regenerative therapy. The drawback of ASCs is that they may serve as stroma for cancer cells and assist tumor progression. It is disquieting that ASCs frequently undergo genetic and epigenetic changes during their in vitro propagation. In this study, we describe the polyploidization of murine ASCs and the accompanying phenotypical, gene expressional and functional changes under long term culturing.

**Methods:**

ASCs were isolated from visceral fat of C57BL/6 J mice, and cultured in vitro for prolonged time. The phenotypical changes were followed by microscopy and flow cytometry. Gene expressional changes were determined by differential transcriptome analysis and changes in protein expression were shown by Western blotting. The tumor growth promoting effect of ASCs was examined by co-culturing them with 4 T1 murine breast cancer cells.

**Results:**

After five passages, the proliferation of ASCs decreases and cells enter a senescence-like state, from which a proportion of cells escape by polyploidization. The resulting ASC line is susceptible to adipogenic, osteogenic and chondrogenic differentiation, and expresses the stem cell markers CD29 and Sca-1 on an upregulated level. Differential transcriptome analysis of ASCs with normal and polyploid karyotype shows altered expression of genes that are involved in regulation of cancer, cellular growth and proliferation. We verified the increased expression of Klf4 and loss of Nestin on protein level. We found that elevated production of insulin-like growth factor 1 by polyploid ASCs rendered them more potent in tumor growth promotion in vitro.

**Conclusions:**

Our model indicates how ASCs with altered genetic background may support tumor progression.

**Electronic supplementary material:**

The online version of this article (10.1186/s12885-018-4781-z) contains supplementary material, which is available to authorized users.

## Background

Stem cell-based therapies for treatment of a wide range of diseases are becoming increasingly important. Currently, adult stem cells appear to be the safest and most reliable source of stem and progenitor cells for tissue engineering and repair. The most widely used cell types for stem cell-based therapies are the mesenchymal stem cells (MSCs). MSCs are multipotent progenitors; they have the ability to differentiate into cartilage, bone, connective, muscle, and adipose tissue [1]. Until recently, the main source of MSCs was the bone marrow, however, adult adipose tissue might be a better alternative source of MSCs, as these can be harvested using minimally invasive procedures. Adipose tissue contains a biologically and clinically intriguing heterogeneous cell population called stromal vascular fraction (SVF) from which the adipose stem cells (ASCs) can be extracted in large quantities and can easily be cultured and expanded. Studies, based on in vivo animal models, have demonstrated that ASCs show the main characteristics of MSCs and might be as efficient and safe for stem cell-based therapies [2]. As a result of these research achievements, various clinical trials have been permitted worldwide. However, detailed analysis revealed chromosomal instability of in vitro expanded stem cells, either of human or murine origin [3–5]. Although aneuploidy has been suggested to be associated with cancer, no transformation of ASCs was observed, and aneuploid ASCs were incapable of tumor formation in immunodeficient mice [3]. On the other hand, both MSCs and ASCs have been shown to incorporate into tumor stroma and promote tumor growth by direct cell-cell contact or paracrine factors [6–8], which questions the safety of stem cell therapies. These findings prompted us to investigate whether ASCs with abnormal karyotype acquire intense tumor promoting activity.

Murine MSCs and ASCs are particularly sensitive to culturing conditions, such as oxidative stress or lack of extracellular matrix, leading to polyploidization and uncontrolled growth [9, 10]. Therefore, they are good models for analyzing the tumor-promoting effect of stem cells with unstable genome. In this study we have isolated and cultured murine ASCs, and analyzed them in long-term culture. We describe the phenotypical, gene expressional and functional changes of ASCs, which accompany the chromosomal abnormalities. We demonstrate that ASCs with abnormal chromosomal number enhance the proliferation of breast cancer cells via insulin-like growth factor 1 (IGF1) production.

## Methods

### Isolation and culturing of vASCs

Since mice of similar age are unique in composition and physiology of their bodies including the amount of body fat, we sacrificed 3–5 mice (4 months old, males and females, C57BL/6 J strain, JAX) for each ASC isolation experiment, to get the best statistical approximation to investigate an average population of ASCs. Visceral fat tissues (epididymal from males or periuterine and periovarian fat from females) were collected into PBS and carefully cut into small pieces (less than 1 mm). Fat tissue was collected by centrifugation at 340×*g* for 10 min and resuspended in DMEM/F-12 (Gibco) supplemented with Penicillin/Streptomycin (Gibco) and 1 mg/ml Collagenase from Clostridium histolyticum (Sigma). Collagenase treatment was done at 37 °C for 1 h with shaking at 200 rpm. Cells were collected by centrifugation at 340×*g* for 10 min. Cell pellet was resuspended in StemXVivo® MSC Expansion Media (R&D Systems, RD-CCM004). Viable cell number was determined by using a BioRad TC counter device. Cells were seeded in cell culture dishes and cultured at 37 °C and 5% CO_2_. Non-adherent cells were removed by sequential changes of the medium twice a week. Cells were passaged using TrypLE-Express (Thermo Fisher Scientific) when they reached about 90% confluency. After 2 passages, cells were cultured further in DMEM/F12 supplemented with L-glutamin (Gibco), Penicillin/Streptomycin, 10% fetal calf serum (FCS, Gibco) and 5% horse serum (HCS, Gibco). For establishing ASC.B6 cell line we cultured ASCs in DMEM/F12 supplemented with L-glutamin, Penicillin/Streptomycin, 10% FCS and 5% HCS until spontaneously immortalized cells appeared and populated the cell culture area. Subsequently, we further cultured these cells by continuous passaging. We regularly prepared frozen stocks of ASC.B6 cells, and stored them in liquid nitrogen. Mesenchymal stem cells from bone marrow, thymus, aorta wall and spleen were isolated and cultured in the laboratory of Prof. Ferenc Uher (National Blood Service, Budapest, Hungary) as described [11]. For preparation of conditioned media, confluent cell cultures were kept in serum-free DMEM/F12 for 48 h, and then the supernatants were harvested and centrifuged for 10 min at 300×*g* to remove cell debris. The supernatants were aliquoted and kept at − 80 °C until use.

### Differentiation of vASCs

vASCs were differentiated into adipocytes, osteocytes and chondrocytes by using the Mouse Mesenchymal Stem Cell Functional Identification Kit (R&D Systems, SC010), according to manufacturer’s instructions. To demonstrate chondrogenesis, high-density pellet cultures were embedded in Tissue-Tek O.C.T. compound (Sakura Finetek), frozen in liquid nitrogen and cryosectioned (6 μm thickness). To analyze the secretion of cartilage proteoglycans, sections were stained with alcian blue 8GS (Roth).

### Detection of senescence-associated beta-galactosidase (SA-βgal) activity

Fifty thousand cells/well were seeded onto 6-well plate and cultured for 24 h. Then the cells were washed twice with PBS and fixed with 4% formaldehyde in PBS for 5 min. After PBS washing, the samples were stained for detecting SA-βgal activity as described in [12]. Blue color was detected after 16 h.

### Cytology experiments

Cells were blocked in mitosis by adding 5 μg/ml colchicine for 5 h. Cells were treated with 75 mM KCl solution, then fixed and washed three times in methanol: acetic acid (3: 1) solution at -20 °C. Suspension of chromosomes and nuclei was dropped, fixed and dried onto microscope slides. Slides were mounted in Vectashield (Vector Laboratories) including 4′,6-diamidino-2-phenylindole (DAPI) (0.5 μg/ml).

### Microscopy

Pictures of DAPI-stained chromosomes or differentiated cells were photographed under a Zeiss Axiovision Z1 fluorescent microscope with 20× and 63× objectives, and analyzed with Axiovision 4.8 software. Morphology of vASC cultures and ASC.B6 cells was checked by Olympus CKX41 inverted light microscope with 4× objective and photomicrographs were taken with an Olympus Camedia C-5060 camera (Olympus Holding Europa GmbH).

### Flow cytometry for DNA-content

Determination of DNA-content of cells was conducted according to a cell cycle analysis protocol. Briefly, the cells at different passages were collected, washed in PBS and incubated with the DNA-staining solution (0.1% Triton X-100, 0.1% sodium citrate, 10 μg/ml RNase and 10 μg/ml propidium-iodide in PBS) for 15 min at room temperature, then analyzed with FACSCalibur flow cytometer using CellQuest software (Becton Dickinson). Freshly isolated splenocytes from a C57BL/6 J mouse were used as controls for diploid status and for setting the gates (Additional file 1: Figure S1).

### Flow cytometry for cell surface markers

Visceral ASCs at different passages or ASC.B6 cells were resuspended in FACS-buffer (PBS supplemented with 2% FCS, 2 mM EDTA and 0.1% sodium azide) at a concentration of 10^6^ cells/ml. For each marker, 90 μl of the cell suspension was incubated with 10 μl of antibody from the Mouse Mesenchymal Stem Cell Marker Antibody Panel (SC018) from R&D Systems. The kit contained antibodies to Sca-1, CD106, CD105, CD73, CD29, CD44, CD11b, and CD45. This reaction was incubated for 2 h on ice, and then the samples were washed twice in 1 mL of FACS-buffer. The secondary developing reagent, anti-rat IgG-TRITC (Millipore) was added according to the manufacturer’s instructions. Following incubation for 30 min on ice, we washed the samples twice in 1 mL of FACS-buffer and performed the FACS analysis with a Guava PCA FACS machine (Millipore).

### Transcriptome analysis

Total RNA samples were isolated from ASC.B6 and vASC sample replicates with the Zymo Research Quick-RNA Miniprep Kit (R1054). We typically used 3-9 × 10^6^ cells for each preparation. Cells were lysed in 700 μl lysis buffer. The manufacturer’s protocol was followed through the process. Total RNA sample was resuspended in 40 μl water that was provided by the Kit. Whole transcriptome cDNA library preparation and analysis was based on the SOLiD Total RNA-Seq Kit (Applied Biosystems). RNA sample quantity and quality was monitored using the Agilent TapeStation 4200 instrument and Eukaryote Total RNA Assay Kit (Agilent, 5067–5576) during RNA processing. RNA was DNAse treated, rRNA depleted (RiboZero Kit, Rat_Mouse Kit, Illumina, MRZH11124), RNAseIII fragmented, hybridized and ligated to oligonucleotide adaptor mix. Reverse transcription was performed with the ArrayScript RT enzyme (Thermo Fisher Scientific, AM2049). cDNA samples were purified and size selected on AMPure XP Beads (Agencourt, A63882) in two rounds, amplified with AmpliTaq (Thermo Fisher Scientific, 4,398,818) and the amplified library was further purified on AMPure XP Beads (Agencourt, A63882) with PureLink PCR Micro Kit (Thermo Fisher Scientific, K310250). Size distribution of the amplified cDNAs were checked by the High sense DNA Kit (Agilent, 5067–1504). Library quantification was done by the SOLiD Library qPCR Standard Kit (Applied Biosystems). In the final library amplification step a full scale emulsion PCR was performed utilizing a SOLiD EZ Bead Emulsifier (Applied Biosystems, 4,448,419). Sequencing was performed on a SOLiD 4 platform (Applied Biosystems). Unmodified, 50-nt long single-end reads were filtered and aligned to the reference mouse genome (Ensembl “GRCm38.77”, 2014). Filtering, mapping and transcript quantification was performed with CLC Genomics Workbench version 8.5.1 using the legacy “RNA-Seq” pipeline with these parameters: maximum number of mismatches = 2, unspecific match limit = 10, use colorspace encoding = yes, use strand specific assembly = no, strand = forward, exon discovery = yes, minimum number of reads = 10, minimum length of putative exons = 50, expression level = genes, expression value = number of reads mapped to the gene. Two independent experiments were done for each sample. Raw gene expression data was imported into R (version 3.3.2, R Core Team, 2015). Subsequently, “calcNormFactors” function from package “edgeR” (version 3.14.0, [13]) was used to perform data normalization based on the “trimmed mean of M-values” (TMM) method [14]. Unsupervised cluster analysis (with Euclidean distance calculation and complete-linkage clustering) was carried out on the group of significantly differentially expressed genes using “heatmap.2” function from R package “gplots”. List of differentially expressed genes in ASC.B6 versus vASC comparison was generated with edgeR functions “exactTest” and “topTags”. Volcano plot showing statistically significant gene expression changes was also created in R. The functional enrichment analyses were generated through the use of Ingenuity Pathway Analysis (Qiagen) system [15] to test whether the observations in differential gene expression can be associated with cellular proliferation or tumor growth.

### Western blotting

The cells were harvested by TrypLE-Express treatment, then washed twice in PBS and counted. Cell concentration was adjusted with sample loading buffer to 5 × 10^6^ cells/ml, boiled for 5 min and then vortexed for 1 min. Boiling and vortexing was repeated 3 times. Samples were centrifuged for 1 min at 13000×*g*, and equal amounts (lysate of 10^5^ cells) were run on an SDS-PAGE. Proteins from conditioned media were precipitated with 10% trichloroacetic acid for 5 min on ice, and then centrifuged for 5 min at 13000×*g.* The pellet was washed with ice-cold acetone and dissolved in sample loading buffer, then run on an SDS-PAGE. The proteins were transferred to polyvinylidene difluoride membranes (Immobilon-P PVDF, Millipore). The membranes were blocked with 5% skim milk or 3% gelatin from cold-water fish skin (Sigma) in PBS for 1 h at room temperature, and then incubated with various primary antibodies overnight at 4 °C. Antibodies used were the following: polyclonal goat anti-mouse Klf4 (R&D Systems, AF3158), polyclonal goat anti-Nestin (Santa Cruz Biotechnology, sc-21,248), polyclonal rabbit anti-mouse IGF1 (Abcam, ab9572). After washing and incubating with HRP-conjugated secondary antibodies (rabbit anti-goat Ig-HRP, Sigma; or swine anti-rabbit Ig-HRP, DAKO) for 1 h at room temperature, the immunoreactive proteins were visualized using WesternBright ECL HRP substrate (Advansta), and the chemiluminescence signal was detected with Odyssey Imaging System (LI-COR Biotechnology). The amount of loaded proteins was tested with rabbit anti-β actin antibody (Abcam, ab8227), followed by anti-rabbit Ig-HRP. To re-probe with different antibodies, the membranes were stripped in stripping buffer (Re-Blot Plus Strong, Millipore) for 15 min at room temperature.

### Proliferation test

4 T1 mouse breast cancer cells were starved in DMEM/F12 supplemented with 0.5% FCS for 24 h, then washed and plated onto 24 well plate at 5 × 10^4^ cells/well in DMEM/F12 containing 2% FCS, in which cells survive but do not proliferate. For co-culture, ASC.B6 cells or vASCs at P3 were seeded in Transwell inserts with 0.4 μm pore size (Corning Costar), at a 4 T1: ASC ratio of 2.5: 1, i.e. 2 × 10^4^ cells/insert. As positive control for cell proliferation, 4 T1 samples were cultured in DMEM/F12 containing 10% FCS. For blocking IGF1, neutralizing antibody (polyclonal rabbit anti-mouse IGF1, Abcam, ab9572) was added at a concentration of 2 μg/ml in lower chambers. All samples were in triplicates. 4 T1 cells were harvested after 48 h, and the cell number was determined by counting cell concentration with Guava PCA flow cytometer.

### Statistical analyses

Mean and SD were determined with Microsoft EXCEL software from the results of at least three independent experiments, each conducted in triplicate samples, unless indicated otherwise in the figure legends. For statistical analysis, pairwise comparisons of experimental groups were carried out using t-test (set at *P < 0.05, **P < 0.01, ***P < 0.001).

## Results

### Characterization of freshly isolated and in vitro cultured adipose tissue-derived stem cells

**Fig. 1:**
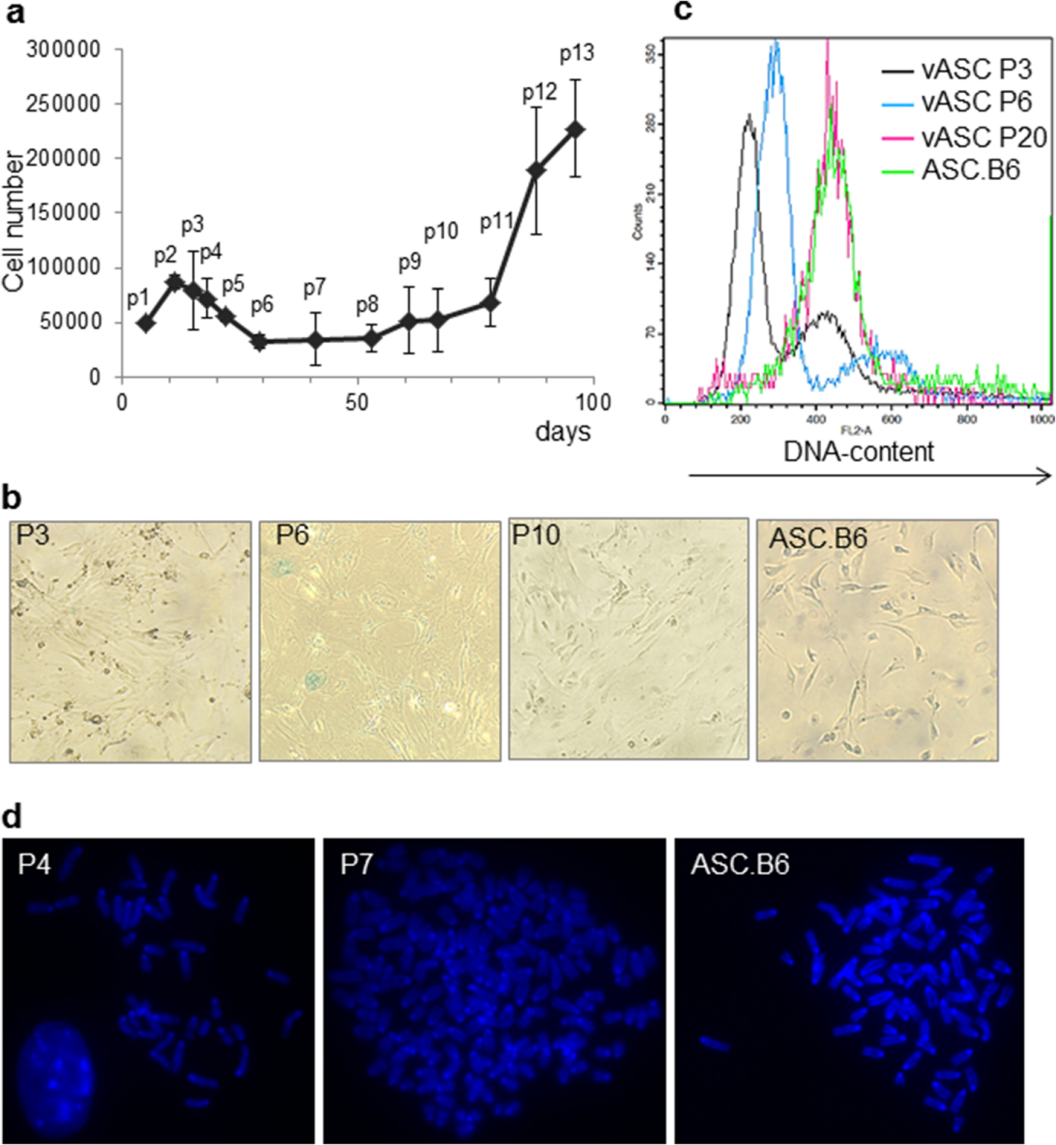
Changes in the proliferation, morphology and ploidy of vASCs under prolonged in vitro culturing. **a** Fifty thousand vASCs were plated and cultured in triplicate samples. Cells were passaged when the culture reached confluency and the living cell number of vASCs was determined with trypan blue staining and counting with BioRad TC10 counter device. The graph shows the average ± SD of living cell numbers in three parallel samples. The x-axis indicates days in culture from the initial plating, and the measuring points are referred as p1 to p13. A representative of 3 independent experiments is shown. **b** vASCs at passage numbers 3, 6 and 10 and ASC.B6 cell line was stained for SA-βgal activity for 16 h and then the blue staining was detected with inverted light microscope. **c** The DNA-content of vASCs at passage numbers 3, 6 and 10 and of ASC.B6 cell line was determined by propidium-iodide staining and flow cytometric analysis. **d** Metaphase chromosome spreads were made from colchicine-blocked vASCs at passage numbers 4 and 7, and ASC.B6. Chromosomes were DAPI stained and counted using fluorescent microscope

ASCs are an exceptionally good source for cell and gene therapy applications, but the reports on their chromosomal instability warn on their possible tumorigenic effect. We aimed to investigate this possibility in mouse model experiments. We isolated and cultured ASCs from visceral adipose tissue of 4 months old mice (C57BL/6 J strain, JAX). When we compared separate isolations of visceral ASC (vASC) populations, we observed that certain features of vASCs showed strong and unmistakable changes. We noticed that the proliferation of vASCs slowed down and the cell number decreased after 5–6 passages, which meant approximately 3 weeks (18–22 days) in vitro culturing (Fig. 1a). The cells also changed morphology under long-term culture conditions, since they became enlarged and flattened, showing signs of senescence (Fig. 1b), however, only few cells exhibited senescence-associated β-galactosidase activity (Fig. 1b). Surprisingly, some of the cells overcame the cellular senescence-like state and continued to proliferate, leading to cell culture expansion after 8–10 passages (> 50 days in vitro), and obtaining elongated morphology again (Fig. 1a and b). In parallel, flow cytometric analysis showed that the DNA content of cells increased at P6 compared to the diploid cells at P3 (Fig. 1c), suggesting that the cells started to alter their diploid chromosome number and the proportion of cells in tetraploid or higher ploidy stage increased (Fig. 1c). Cells at higher passage number (P20) were predominantly hypotetraploid (Fig. 1c). We also checked the average chromosome number of the cells by microscopy. Fig 1d shows that vASC at P4 had 40 chromosomes, but the chromosome number in cells at P7 could even reach 163. We found that after P7, vASCs consistently demonstrated a hypotetraploid karyotype, with average of 73–76 chromosomes. As cells after 8–10 passages started to grow intensively, we suspected that clones of hypotetraploid cells became immortalized. We established a continuously proliferating adipose-tissue stem cell line, named ASC.B6, cultured in vitro for more than 50 passages, having fibroblastoid morphology and hypotetraploid karyotype (Fig. 1b-d). Moreover, we also examined the chromosome number of established MSC lines from various mouse tissues, such as bone marrow, spleen, aorta and thymus (Additional file 2: Figure S2). These cell lines also had hypotetraploid karyotype, indicating that hyperploidization is not restricted to adipose-derived stem cells.

**Fig. 2:**
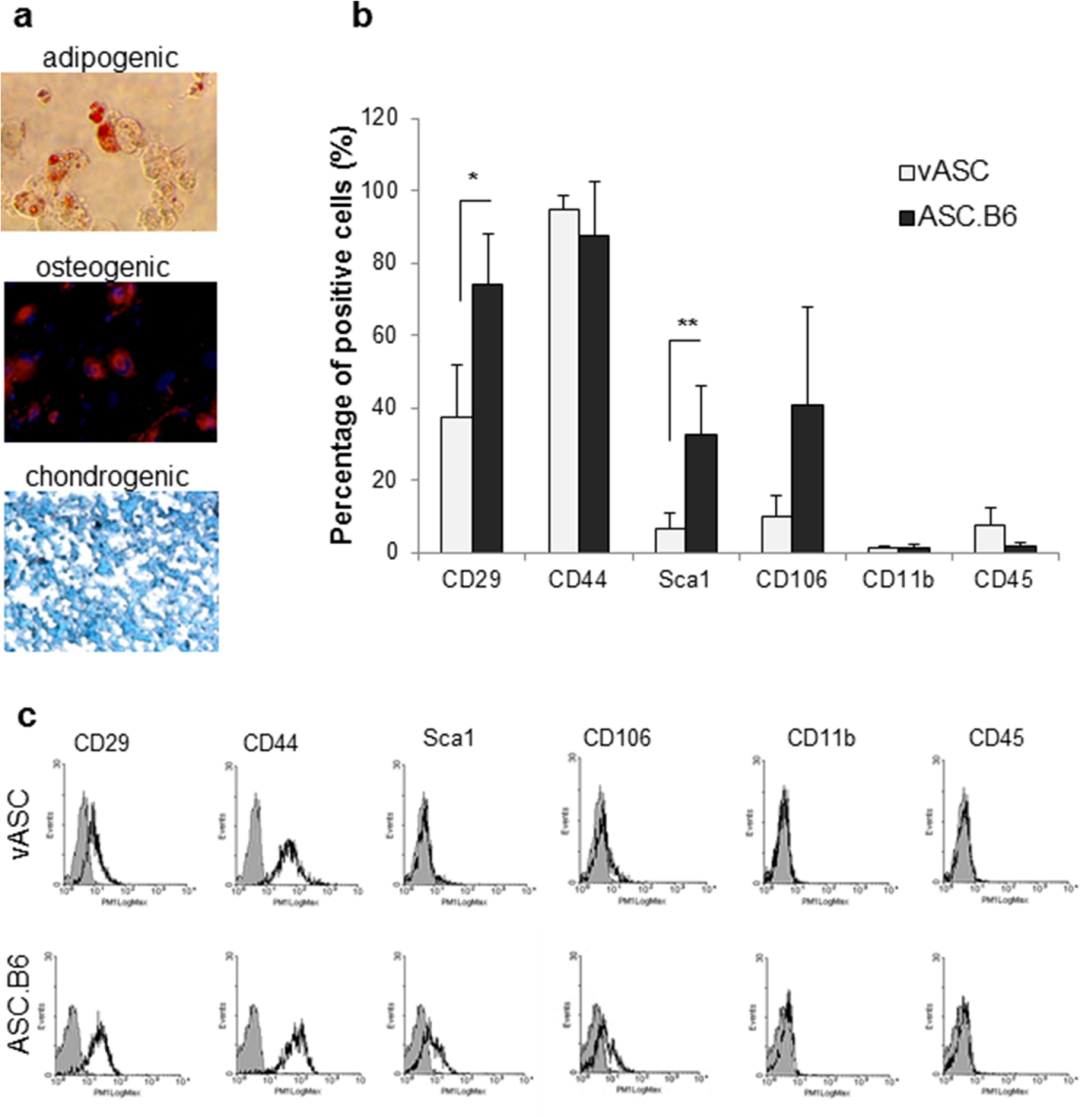
Characterization of the ASC.B6 cell line. **a** In vitro adipogenic, osteogenic and chondrogenic differentiation of ASC.B6 cells were detected with Oil Red, anti-osteopontin antibody and Alcian Blue staining, respectively. **b** Cell surface markers of vASCs at passage 3 and ASC.B6 cells were detected by flow cytometry and the percentage of positive cells were determined. The bars show the mean ± SD from 3 independent experiments, the statistical analysis was t-test with *P*-values set at: *P < 0.05, **P < 0.01. **c** Representative pictures of cell surface marker analysis by flow cytometry

Since ASC.B6 cells showed an abnormal karyotype, our next question was whether these cells still behaved as ASCs. A common feature of ASCs is the ability to differentiate into mesenchymal cell types by default: adipocytes, chondrocytes and osteocytes. We found that our cell line retained its differentiation capability and hence the stem cell property (Fig. 2a). On the other hand, we also examined these cells for the presence of the most common cell surface markers of adipose-derived stem cells [16], taking into account that the positive markers show different distribution between various species, and even between different mouse strains [17, 18]. We detected changes in the representation of cell surface markers on ASC.B6 compared to vASCs (Fig. 2b and c). The expression of CD29 (integrin β1) and stem cell antigen-1 (Sca-1, Ly6A) significantly increased, and the proportion of CD106 (vascular cell adhesion molecule 1, VCAM-1) positive cells also augmented in ASC.B6 cell culture (Fig. 2b and c). In addition, both vASCs and ASC.B6 highly expressed the stem cell marker CD44, but not the hematopoietic markers, CD11b and CD45 (Fig. 2b and c). None of the cells expressed other stem cell markers, such as CD73 (ecto-5′-nucleotidase) or CD105 (endoglin) (data not shown).

### Gene and protein expressions change during the in vitro culturing of ASCs

**Fig. 3:**
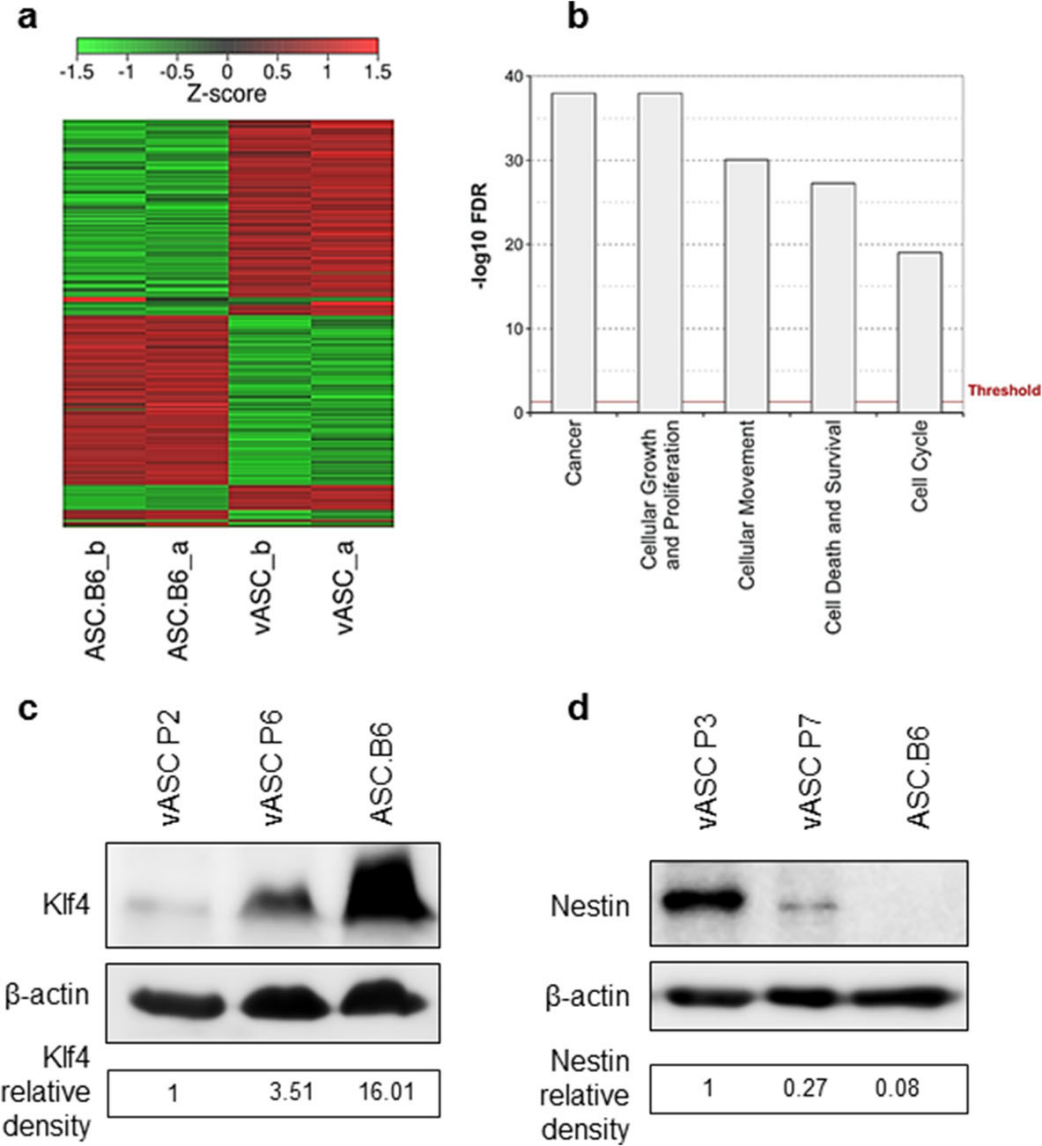
Gene and protein expression change during the in vitro culturing of ASCs. **a** Heatmap represents the hierarchical clustering of the 2395 genes expressed differentially (− 2× > FC > 2× and FDR < 0.05) in the RNA-seq experiment. The investigated cell types are indicated at the bottom of the chart. Both groups consist of biological replicate pairs (a, b) with highly similar pattern, in contrast, vASC pair demonstrates strikingly different pattern compared to ASC.B6 pair. **b** Functional enrichment analysis done by IPA for the identification of biological functions and diseases that were most profoundly represented by the differentially expressed genes. Only carcinogenesis associated categories are presented, all of which are highly affected. Right-tailed Fisher’s exact test was used to calculate a *p*-value. False discovery rates (FDR) were generated based on the Benjamini-Hochberg corrected *p*-values. **c** Klf4 protein expression was analyzed with Western blotting in total cell lysates of vASCs harvested at P2 or P6 and ASC.B6 cells. The Klf4 signals were normalized to β-actin, and divided with the value of vASCs at P2. **d** Nestin protein expression was analyzed with Western blotting in total cell lysates of vASCs harvested at P3 or P7 and ASC.B6 cells. The Nestin signals were normalized to β-actin, and divided with the value of vASCs at P3. The pictures show a representative of at least 3 independent experiments

Based on phenotypic observation we assumed that changes in the karyotype led to altered gene expression, therefore we performed a differential transcriptome analysis to get a more detailed picture. DNA-free total RNA samples were purified from vASCs at P3 or ASC.B6 cultures, and submitted to high throughput RNA sequencing and bioinformatic processing. Two thousand, three hundred and ninety-five genes demonstrated at least 2-fold (log_2_ FC > = 1) statistically significant (FDR < 0.05) gene expression changes in ASC.B6 compared to vASC cells (Fig. 3a and Additional file 3: Figure S3). It was also shown that among others cancer, cellular growth, proliferation and cellular movement functional gene annotation categories were markedly overrepresented in gene set differentially expressed between ASC.B6 cell line and vASCs (Fig. 3b) at low passage numbers, suggesting that acquirement of polyploidy under long term culturing essentially changed cell physiology. The results of transcription analysis were in some cases in good correlation with the flow cytometry results, as the transcription of the stem cell marker Sca-1 and the adhesion molecule CD106 increased in ASC.B6 (Fig. 2b and Table 1). However, the cell surface level of CD29 increased despite there was no change in its transcription. Moreover, the presence of CD44 did not decrease significantly in contrast to its gene expression level (Fig. 2b and Table 1), suggesting differential regulation mechanisms in protein turnover. The elevated expression of CD29 and Sca-1 indicated that ASC.B6 cells shifted to a cancer stem cell phenotype [19, 20]. Focusing on the presence of pluripotency markers thereafter, we observed increased gene expression of Krüppel-like factor 4 (Klf4) and N-myc, while Nestin, a putative marker of cancer stem cells [21], decreased (Table 1). No changes were detected in the transcription of other embryonic stem cell markers, such as Oct4 (Pou5F1), Nanog and SSEA-1 (Fut4). The protein expression of Klf4 and Nestin was confirmed with Western blotting (Fig. 3c and d), and the results were consistent with the transcriptome analysis. Moreover, the protein level gradually changed with ploidy changes, showing no expression of Klf4 in vASC at P2, low level at P6, and the highest expression in ASC.B6 (Fig. 3c). We detected Nestin protein expression at P3 in vASCs, which decreased at P7, then diminished in ASC.B6 (Fig. 3d).

**Table 1:** Differential expression of selected genes in ASC.B6 vs. vASC P3

Group	Gene name	logFC	FDR	Expression change ASC.B6 vs. vASC P3
Cell surface markers	Ly6a (Scal)	2.25	1.631E-13	↑
	Vcaml (CD106)	1.29	3.471E-05	↑
	Itgbl (CD29)	−0.17	1.000E + 00	–
	Cd44	−1.39	1.453E-06	↓
Pluripotency and stemness markers	Klf4	2.22	6.115E-11	↑
	Mycn	1.98	1.112E-04	↑
	Pou5F1 (Oct 4)	−0.88	1.000E + 00	–
	Nanog	−0.77	1.000E + 00	–
	Fut4 (SSEA-1)	−0.63	1.000E + 00	–
	Nes (Nestin)	−1.91	9.598E-08	↓
Tumor growth promoting factors	Igf1	1.64	3.344E-07	↑
	Egf	0.03	1.000E + 00	–
	Hgf	0.14	1.000E + 00	–
	Ngf	−0.58	4.889E-01	–
	I16	− 0.45	9.772E-01	–
	Tgfb1	−3.75	3.719E-10	↓
	Fgf2	−1.56	6.540E-06	↓

### ASCs with abnormal karyotype promote tumor-cell proliferation

**Fig. 4:**
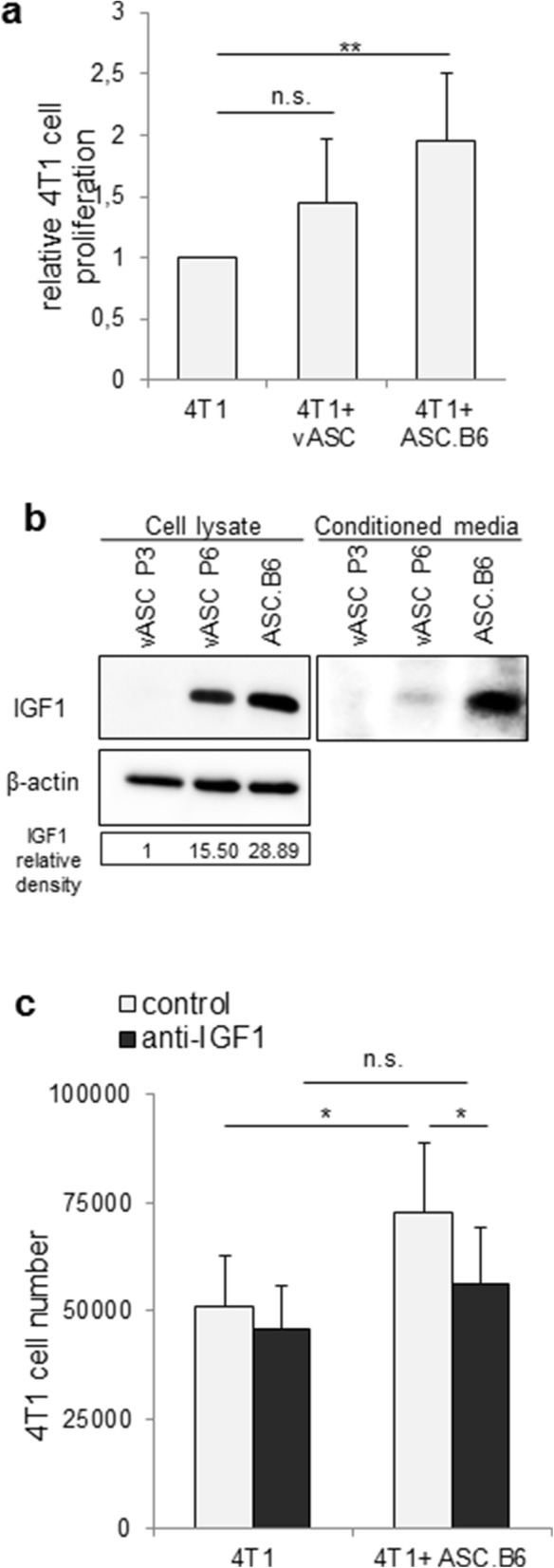
ASCs affect tumor growth. **a** Proliferation of 4 T1 cells in the presence of vASC P3 or ASC.B6 at a ratio of 2.5:1, separated in Transwell inserts. The bars show the mean ± SD from 3 independent experiments, the statistical analysis was t-test with *P*-values set at: **P < 0.01. **b** IGF1 protein expression was analyzed with Western blotting in total cell lysates and conditioned media of vASCs harvested at P3 or P6 and ASC.B6 cells. The IGF1 signals from cell lysates were normalized to β-actin, and then divided with the value of vASCs at P3. The picture shows a representative of at least 3 independent experiments. **c** Neutralizing antibody against IGF1 (2 μg/ml) inhibited the proliferation of 4 T1 cells in the presence of ASC.B6 in Transwell inserts. The bars show the mean ± SD from 3 independent experiments, the statistical analysis was t-test with *P*-values set at: *P < 0.05

ASCs may serve as a source of tumor stroma, hence we examined if cultured vASCs contributed to tumor growth. We co-cultured 4 T1 murine breast cancer cell line with vASC P3 or ASC.B6 in Transwell inserts with 0.4 μm pores. This experimental setup allows free diffusion of soluble growth factors, while it enables the correct distinction and counting of different cell types. Both ASC cultures enhanced the proliferation of 4 T1, however, ASC.B6 was more potent (Fig. 4a). Looking over the results of transcriptome analysis we found that most of the tumor-promoting growth factors [22], such as epidermal growth factor (EGF), hepatocyte growth factor (HGF), nerve growth factor (NGF) or interleukin 6 (IL-6) were expressed at similar level in ASC.B6 and vASC (Table 1). Moreover, expression of transforming growth factor β1 (TGFβ1) and fibroblast growth factor 2 (FGF2) even decreased in ASC.B6, opposing to the increased tumor growth promoting effect (Table 1). In contrast, IGF1 transcription increased in ASC.B6 compared to vASC. Western blotting experiments confirmed that the IGF1 protein expression was upregulated in vASC at P6 and in ASC.B6 cells, and it was secreted to cell culture media (Fig. 4b). Moreover, neutralization antibody against IGF1 significantly decreased the tumor growth promoting effect of ASC.B6, suggesting that IGF1 production contributed to the tumor growth-promoting effect of ASC.B6 (Fig. 4c).

## Discussion

Murine stem cells have been reported to undergo immortalization with concomitant chromosomal abnormalities during prolonged in vitro culture [23]. Most studies have tried to optimize culturing conditions, such as lowering oxygen concentration (2% vs. 20% oxygen), coating the surface with extracellular matrix, adding antioxidants to cell culture media and testing serum supplements from a variety of species [9, 24]. In most reported cases, mesenchymal stromal cells were isolated from bone marrow [24, 25], but the phenomenon was similar for stromal cells from other organs, such as adipose tissue [9, 10]. These abnormalities were also shown for other cell types as well, for example the epithelial cells [26]. Chromosome number changes are usually associated with enhanced proliferation and longevity, indicating that they have beneficial effects in special conditions. Indeed, over half of the mature hepatocytes in mice and humans are aneuploid, and it was proposed that this mechanism evolved to generate genetic diversity and permits adaptation of hepatocytes to oxidative stress caused by xenobiotic or nutritional injury [27]. We have found that under standard in vitro culturing conditions (i.e. normoxia and xenogen serum), which meant stress for freshly isolated cells, adipose-tissue derived stromal cells rapidly underwent polyploidization. Moreover, established stromal cell lines from mouse bone marrow, spleen, thymus and aorta also showed odd chromosome numbers. Aneuploidy has been also detected in prolonged cultures of human adipose tissue-derived stromal cells, which increased dramatically in senescent cultures [3]. We hypothesize that such changes may occur in vivo as well, as a consequence of a locally higher concentration of reactive oxygen species or inflammatory mediators, which are typical for chronic inflammation and tumor microenvironment [28].

We detected the chromosome number abnormities after 6–8 passages, accompanied with phenotypic, gene expressional and functional changes. When cell surface markers were compared, we found the greatest difference in the expression of Sca-1. Sca-1 is a member of the Ly6 family of glycosyl phostidylinositol (GPI)-anchored cell surface proteins, originally used for the enrichment of murine hematopoietic stem cells, but it is also expressed by stem, progenitor, and differentiated cell types in a wide variety of tissues and organs [20]. Recently, Sca-1 has been found to be a selective marker for prospective isolation of mesenchymal stem cells from murine bone marrow, when used in combination with other positive (platelet-derived growth factor receptor α) and negative markers (CD45, TER119) in flow cytometry [29]. We found that Sca-1 is rarely present on stem cells derived from visceral adipose tissue, but interestingly, the expression of Sca-1 was upregulated in continuously proliferating ASC.B6 cells. The function of Sca-1 has not been determined yet. It is suggested to take part in regulating different signaling pathways by modulating lipid raft composition, although its ligand and the exact signaling machinery have not been revealed. Phenotyping of *Sca-1*^−/−^ mice demonstrated a number of defects associated with tissue resident stem and progenitor cell populations. Sca-1 is supposed to be required for the self-renewal of mesenchymal progenitors and, as a consequence, for osteogenesis and adipogenesis [30]. Sca-1 is also upregulated in various murine cancers, including retinoblastoma, and tumors of the mammary gland and prostate. Moreover, the fibroblastic tumor and mammary adenocarcinoma cells expressing high Sca-1 levels are significantly more malignant than those expressing lower levels [20]. Recently, it has been shown that Sca-1 played a role in maintaining tumorigenicity in a mouse mammary tumor model. Sca-1 acted as a potent suppressor of TGF-β, by inhibiting expression of the TGF-β family ligand GDF10 and binding to the type I TGF-β receptor (TβRI), releasing the cells from TGF-β-mediated growth inhibition [31]. Based on these results, we propose that Sca-1 upregulation in ASC.B6 cells contribute to their proliferative capacity. Checking other stem cell markers, we detected increase of Klf4 on both transcriptional and protein level in parallel with passage numbers. Klf4, a member of the Krüppel-like factor family, is expressed in a wide range of tissues in mammals, and it plays a role in regulating proliferation, differentiation, development, maintenance of normal tissue homeostasis and apoptosis [32]. Klf4 is essential for embryonic stem cells’ self-renewal and maintenance [33], and it is one of the four pluripotency-promoting factors used for the establishment of induced pluripotent stem cells (iPSCs) [34]. Its expression level is often altered in various tumors [35], and it plays critical role in the maintenance of cancer stem cells, e.g. in breast and colon cancers [36, 37]. The striking increase of Klf4 in ASC.B6 cells suggests that these cells maintain their stem cell-like properties under long-term culturing. In contrast, we detected Nestin at mRNA and protein level in early vASC cultures, but its expression decreased with passage number. Nestin has been identified as a class VI intermediate filament protein expressed during development of the central nervous system in neural progenitor cells, and downregulated upon differentiation [38]. Nestin has also been suggested to be a specific marker, which is proper for the identification of bone marrow MSCs [39], and it has also been found in adipose tissue derived stem cells [40]. Moreover, expression of Nestin was reported to be upregulated in most mitotic cells [38], including tumor cells and cancer stem cells [21], and downregulated upon differentiation. This regulation is mediated by Tyr-phosphorylation by cdc2 [41]. In our experiments, Nestin was expressed in vASCs at early passage number (P3), and then decreased at later passages. Although the ASC.B6 cell line actively proliferated, we could not detect Nestin expression either on transcriptional or protein level, suggesting that other regulatory mechanisms may have been responsible. Nestin expression is significantly upregulated by hypoxia, which is mediated by HIF-1α and VEGF [42], while oxidative stress rapidly leads to its degradation in a Cdk5-dependent manner [43], which may be the explanation for downregulation of Nestin in ASC.B6 cultured under normoxic conditions. Further experiments are needed for clarifying the mechanism and role of Nestin loss in ASC.B6 cells.

Adipose tissue-derived stem cells may be a source for tumor stroma [8], and it is plausible that vASCs promote the proliferation of cancer cells. Our results showed that the immortalized cell line had more potent proliferative effect than vASCs with normal karyotype. We found that vASCs produced IGF1 and its protein level increased with passage number. We have also demonstrated that IGF1 contributed to the proliferation of breast cancer cells. IGF1 is a key growth factor of mammary terminal end bud and ductal formation during development; however, it also plays an important role in breast cancer development, progression and metastasis [44]. Primarily, the liver produces IGF1, however, it is also expressed by mammary stromal cells, contributing to mammary gland development in a paracrine manner [45]. The anti-apoptotic and tumorigenic effect of IGF1 is mediated by binding to its cognate receptor, IGF-1R, which is frequently overexpressed in breast cancer. It has been shown that IGF1, which was released by precursor or differentiated adipocytes promoted the proliferation of breast cancer cells in vitro, and a correlation has been found between obesity, fatty acids and IGF1 levels, suggesting that hyperactivity of adipose tissue may contribute to tumorigenesis [46]. Numerous studies suggested that the IGF1 system is a promising candidate in breast cancer therapies. Many strategies targeted the IGF-1R, either with monoclonal antibodies (mAbs) or with receptor tyrosine kinase inhibitors; however, they failed in phase III breast cancer clinical trials. The IGF-1R antibody therapy resulted in hyperglycemia and metabolic syndrome, and the receptor tyrosine kinase inhibitors manifested metabolic toxicities, most likely due to high similarity between IGF-1R and insulin receptor [47]. In contrast, there are only two IGF-1/2 neutralizing mAb in clinical trials currently under investigation [47]. BI836845, mAb targeted against IGF-1/2, showed encouraging results in preclinical models: it reduced the proliferation of human cell lines derived from different cancer types, inhibited tumor growth in a xenograft model and had additive effect in combination with rapamycin [48]; hence, it is now in clinical trial phase II for metastatic breast cancer patients (www.clinicaltrials.gov, NCT02123823). Another anti-IGF1 mAb, MEDI-573 is also in phase II clinical trial currently for late stage breast cancer (www.clinicaltrials.gov, NCT01446159).

Recently, it has been recognized that tumor stromal cells support cancer cells’ chemo-resistance by secreting IGF1 and 2 [49] therefore, finding therapeutic targets on tumor stroma has become an urgent task. There are efforts to block tumor-stromal signaling crosstalk by small molecule inhibitors, for example NT157 that leads to the irreversible elimination of an important IGF-1R signaling protein, the Insulin receptor substrate 1 and 2 (IRS-1/2), and that has pharmacological effect in osteosarcoma and prostate cancer in preclinical studies, but there are no results for breast cancer yet [47].

## Conclusions

ASCs are proposed to serve as stroma for cancer cells and thereby assist tumor growth. Our results show that ASCs with altered genetic background promote tumor progression by upregulating the expression of genes, such as IGF1, which favors carcinogenesis. We propose that mouse adipose tissue-derived stem cells are suitable model to further search for genes responsible for initiating and propagating tumorigenesis and may serve as a preclinical system to study the efficacy of novel therapeutics for targeting tumor stroma.

## Additional files

Additional file1: **Figure S1**. Gating strategy for cell cycle analysis of mouse spleen cells, as diploid control. (TIF 2194 kb)

Additional file2: **Figure S2**. Determination of chromosome number in metaphase spreads of established mesenchymal stem cell by DAPI staining and fluorescent microscopy. **a** MSCs from mouse aorta have an average chromosome number of 57. **b** MSCs from mouse bone marrow have an average chromosome number of 69. **c** MSCs from mouse spleen have an average chromosome number of 76. **d** MSCs from mouse thymus have an average chromosome number of 74. (TIF 2194 kb)

Additional file3: **Figure S3**. Volcano plot displays the fold change (FC) and statistical significance (FDR) values of gene expression differences detected in the RNA-seq investigation of ASC.B6 and vASC cells. Each dot represents an individual gene. Selection thresholds of genes for further clustering and gene enrichment analysis is indicated by horizontal and vertical red dashed lines (FDR < 0.05 and FC < − 2 or FC > 2, respectively). Further analyzed and discussed genes (Igf1, Sca-1, Klf4, Nestin) are highlighted on the plot with specific colors. (TIF 2194 kb)

## Abbreviations

ASCs: Adipose-tissue stem cells
IGF1: Insulin-like growth factor 1
MSC: Mesenchymal stem cells
vASC: Visceral adipose-tissue stem cells
