# Supplementary figures and images for: Increased insulin-like growth factor 1 production by polyploid adipose stem cells promotes growth of breast cancer cells
Source: BMC Cancer. 2018 Sep 5;18:872. doi: 10.1186/s12885-018-4781-z (PMC6126028; doi:10.1186/s12885-018-4781-z)

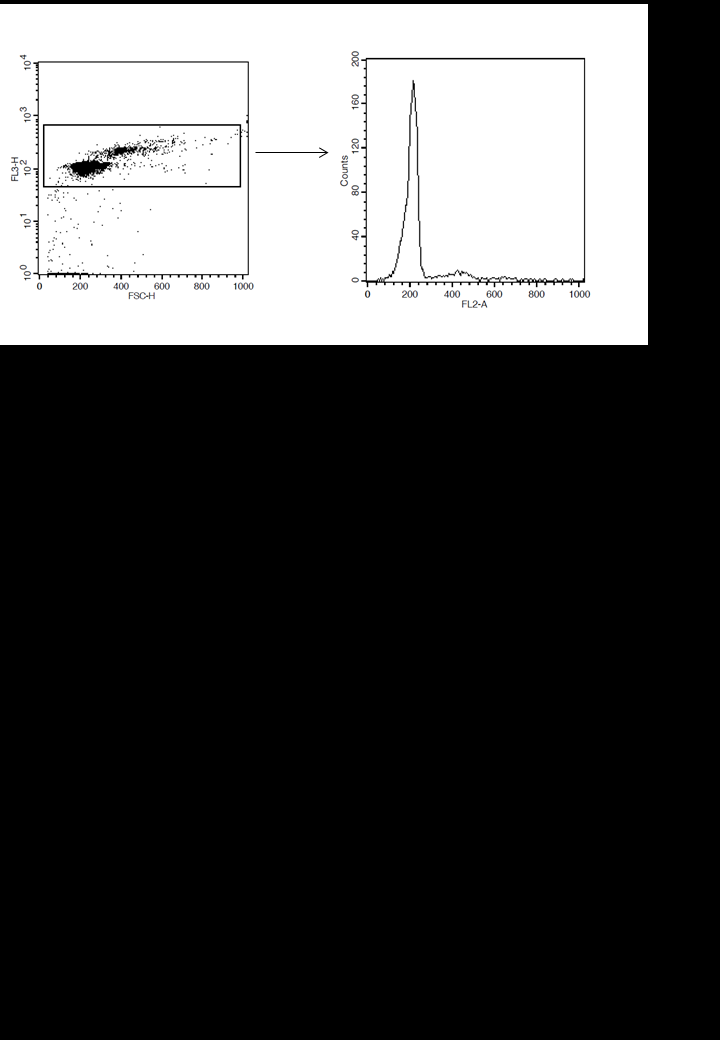

Supplement: Supplementary file 1 — Figure S1. Gating strategy for cell cycle analysis of mouse spleen cells, as diploid control. (TIF 2194 kb) [file 12885_2018_4781_MOESM1_ESM.tif]

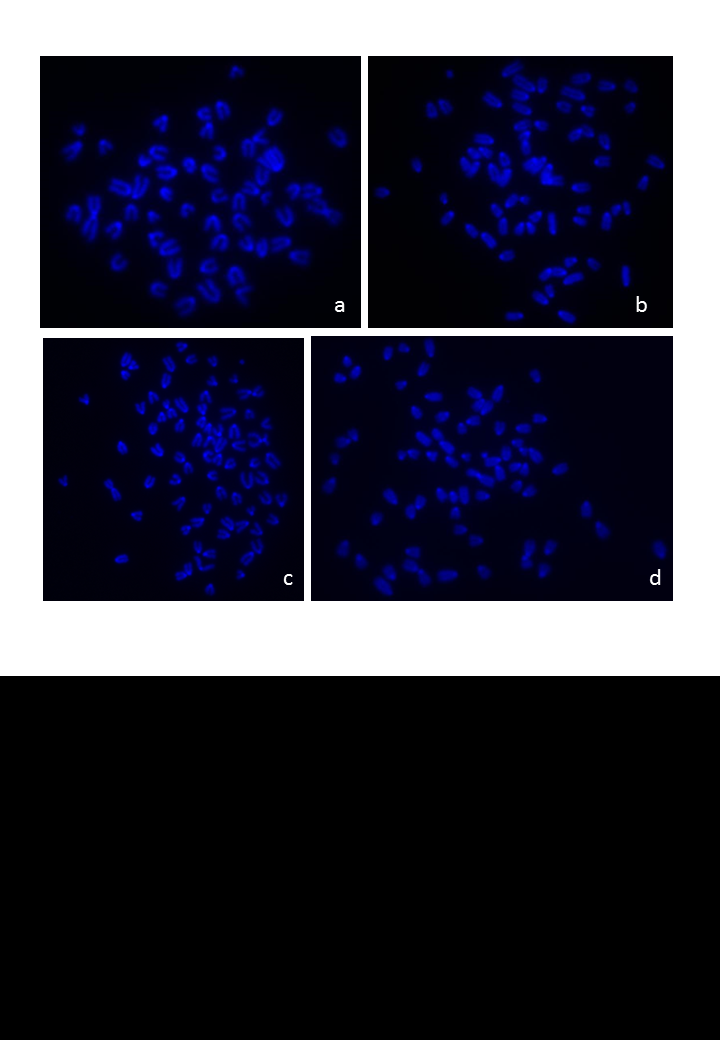

Supplement: Supplementary file 2 — Figure S2. Determination of chromosome number in metaphase spreads of established mesenchymal stem cell by DAPI staining and fluorescent microscopy. a MSCs from mouse aorta have an average chromosome number of 57. b MSCs from mouse bone marrow have an average chromosome number of 69. c MSCs from mouse spleen have an average chromosome number of 76. d MSCs from mouse thymus have an average chromosome number of 74. (TIF 2194 kb) [file 12885_2018_4781_MOESM2_ESM.tif]

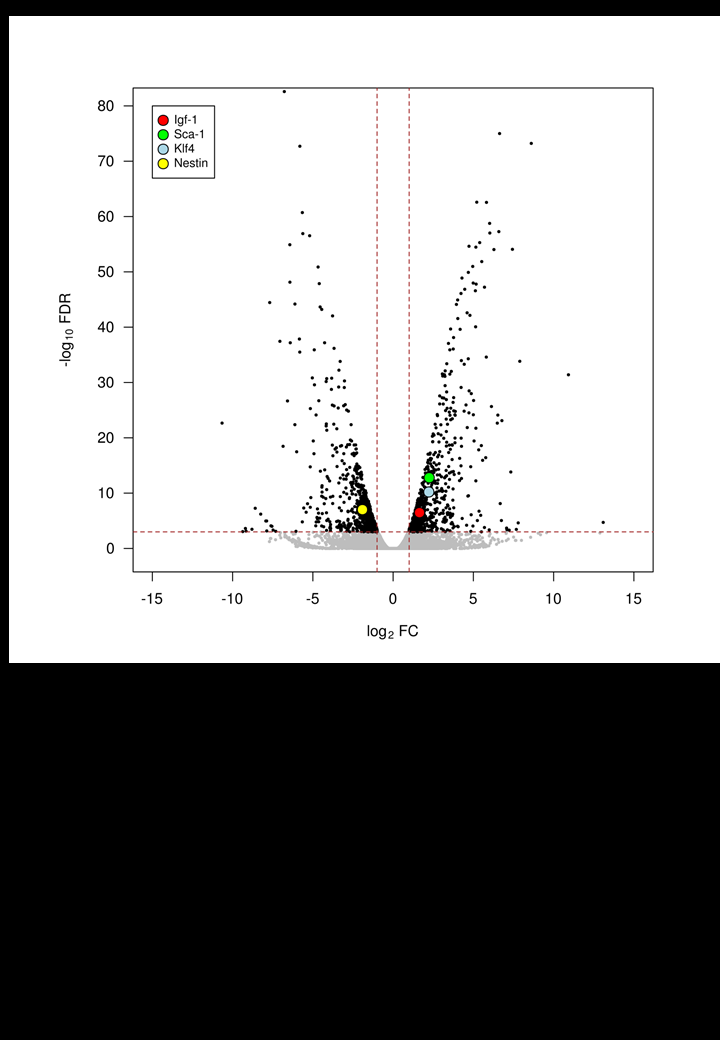

Supplement: Supplementary file 3 — Figure S3. Volcano plot displays the fold change (FC) and statistical significance (FDR) values of gene expression differences detected in the RNA-seq investigation of ASC.B6 and vASC cells. Each dot represents an individual gene. Selection thresholds of genes for further clustering and gene enrichment analysis is indicated by horizontal and vertical red dashed lines (FDR < 0.05 and FC < − 2 or FC > 2, respectively). Further analyzed and discussed genes (Igf1, Sca-1, Klf4, Nestin) are highlighted on the plot with specific colors. (TIF 2194 kb) [file 12885_2018_4781_MOESM3_ESM.tif]
